# The Medicinal Value of Onions

**Published:** 1891-12

**Authors:** M. Sanford


### THE MEDICINAL VALUE OF ONIONS.

I am constrained to make a plea for onions, not as an article of diet particularly, although their value in that form is well known, but as a treatment in cases of lung fever, congestion of the lungs, coughs, colds, etc. Applied as poultices and administered internally in the form of syrup their effect is wonderful. When our little two-year old was so ill with congestion of the lungs last winter our doctor prescribed onions at once, and we think they saved his life. For the benefit of young mothers the following methods of preparing them are given :

For the poultice, peel the raw onions and slice them into a frying-pan containing some good lard. It will probably take three large onions and a level tablespoonful of lard for one poultice ; let them cook slowly, but put no water with them. Have a bag made of thin cheese-cloth, into which put the hot onions and sew up at the end. Make the bag large enough to cover the whole chest and lungs, and in severe cases put a similar poultice on the back between the shoulders. I find it convenient to sew strings to the bags so they may be tied around the little patient's neck, thus preventing their slipping down. The poultices should be changed often enough to keep them warm and several thicknesses of flannel should be put between the poultice and the other clothing.

There is usually a troublesome cough accompanying that may be relieved by a syrup of onions made by slicing raw onions into a bowl in alternate layers with white sugar ; cover and set in the oven until the juice is extracted, but to avoid waste squeeze onions, syrup and all through a strong cloth until no more juice is to be had. Give this syrup at intervals and there is no danger of giving too much, for it will do no harm.

When the poultices are finally removed sew thin slices of fat bacon to a flannel large enough to cover the chest and keep this tied on several days ; then remove the bacon and leave the same greasy flannel over the chest for a week longer, which treatment seems to prevent taking cold easily again.—*M. Sanford.*

